# Unmet needs of patients with cancer in their last year of life as described by caregivers in a developing world setting: a qualitative study

**DOI:** 10.1186/s12904-020-0516-4

**Published:** 2020-01-24

**Authors:** Karen Cox-Seignoret, Rohan G. Maharaj

**Affiliations:** 1 Caura Palliative Care Unit, Caura Hospital, El Dorado, Trinidad and Tobago; 2 The Unit of Public Health and Primary Care, Department of Paraclinical Sciences, The University of the West Indies, St. Augustine, Trinidad and Tobago

**Keywords:** Trinidad, Tobago, Cancer, Palliative care, End-of-life, needs

## Abstract

**Background:**

Palliative care is in its infancy in most of the developing world. We set out to explore the lived experiences of families and caregivers of recently deceased cancer patients in Trinidad and Tobago and to determine the unmet needs of the patients and what recommendations could be derived to improve the current services.

**Methods:**

A phenomenological approach with purposeful sampling was used. Participants were referred by key health professionals. Face-to-face interviews were conducted. Interviews were transcribed verbatim, with analysis and data collection occurring concurrently. Thematic content analysis was used to determine common domains, themes and sub-themes.

**Results:**

Interviews were completed with 15 caregivers. All were spouses or children of the deceased. Ages of the deceased ranged from 43 to 93, the average being 65.5 years. The deceased experienced a variety of cancers including lung, colorectal and oesophageal.

Unmet needs were identified under 4 domains of institutions, community, the family unit and the wider society. Institutional unmet needs were delayed diagnosis and treatment and poor inter-institution coordination. Medical and nursing care failed in the areas of health care providers’ attitudes, pain management and communication. The family unit lacked physical and psychosocial support for the caregiver and financial aid for the family unit. Societal needs were for public education to address myths and cultural beliefs around cancer.

**Conclusion:**

There is need for systemic interventions to improve the care of those dying from cancer in Trinidad and Tobago. Stakeholders need to commit to palliative care as a public health priority, implementing education, planning services and mobilizing community resources.

## Background

Access to palliative care is now considered a basic human right [1, 2]. To uphold this right, the report of the 67th World Health Assembly urges countries to put end of life care on the public health agenda at all levels, to re-define models of service provision appropriate to their culture and needs, and to aim for integration into mainstream healthcare [3].

Global estimates are of 19 million people needing palliative care at the end of life, more than three-quarters of whom inhabit economically deprived countries [4]. In resource-poor countries, palliative care services tend to be scarce [5] and many diseases present late so that the only option is palliation [6, 7].

Trinidad and Tobago (T&T), a twin island state at the southern end of the Caribbean chain, has one of the highest cancer mortality rates in the region [8]. Cancer is the third leading cause of death [9].

Despite the country’s relative affluence, there are significant challenges to health care delivery. Factors influencing the growth of palliative care services have mirrored those in other resource-poor countries, where the initiatives of non-governmental and often faith-based organizations [10, 11] rather than formal needs assessment and a comprehensive national vision, drive development [12].

The local palliative care need has not been well quantified. Unpublished observations are the only source of data. St Elizabeth Health Care in 2005 reported that perhaps as many as 50% of cancer patients are beyond cure, and Chang in 2014 found the one [1] month prevalence of patients qualifying as palliative on an inpatient medical ward to be 23.47%.

Between 2013 and 2018, there has been expansion of local palliative care service capacity. The country has moved from having two twelve [12] bed hospices and one rural community care programme to having additional services in the form of one hospital-based palliative care clinic, one hospital-based consulting service, a home care service in Tobago and Trinidad’s first palliative care unit within the public sector. Despite this progress, however, most end-of-life care continues to be delivered by health workers and institutions with little or no palliative care expertise.

The international literature has identified several domains as being important to patients and families at the end-of-life, among them physical comfort and symptom control, control over decisions and a need for education and for emotional support [13–15]. Not having a prolonged death, being prepared for death and holistic individualized care were also desired [16]. Many desired to care for their loved ones at home [17–19], but the caregiver experience is frequently reported as very challenging [20–24]. Effective communication and appropriate information are domains consistently identified as essential [14, 15, 21, 22, 25–39].

Caribbean literature on end-of-life care is lacking [40], but literature from other low resource settings has identified several barriers to good end-of-life-care. Some of the critical issues cited are the absence of national policies on palliative care and lack of integration of palliative care into mainstream healthcare [41].

For patients and families in low resource settings like the Middle East, Africa and India, these can manifest as insufficient services especially for community care, inadequate training of health professionals, inadequate access to opioids and consequently poor symptom control [11, 41–43].In many African countries, morphine underutilization due to fears on the part of both lay persons and health professionals, have been highlighted [11].

In the limited Caribbean research, Kreitzschitz and Macpherson in Grenada [44] reported suboptimal end-of-life experiences due to poorly managed pain, a result of inadequate prescribing, poor compliance and drug shortages. Other system issues were poor nursing attitudes, lack of privacy, limited access to in-patients and equipment failures. Spence et al. [45] on the larger island of Jamaica found that poor pain control and lack of home care support featured prominently. Financial constraints were cited as the most influential factor prohibiting access to care in their study. Poor communication with health professionals was another major barrier, breeding mistrust that fostered the use of herbal or “bush” medicines. Both the Jamaican and Grenadian studies noted use of herbal remedies and cultural beliefs that required targeted education strategies [44, 45]. Health professionals in Jamaica lamented the absence of palliative care training as well as the lack of governmental commitment to policies and legislation for effective palliative care delivery.

While there is a commonality to many of these themes across countries, it is not known if these end-of-life issues would be mirrored in the Trinidad and Tobago experience. The researcher KCS, although working in general practice at the time of the study, had a special interest in community palliative care and was motivated to investigate the local end-of-life experience.

The objective of this study was therefore to explore for the first time in Trinidad and Tobago, the lived experience of cancer patients in the last year of life, their challenges with the existing health care services and to elicit recommendations for improving care.

## Methods

### Study design

This was a qualitative study utilizing face-to-face interviews with the families or caregivers of patients who died from cancer within the last six [6] to twelve [12] months.

A phenomenological approach was adopted with proxies selected as the source of data. Direct patient interviews would have been preferable, but the fragility of the palliative patient population makes this challenging [46–48] with up to 35% of the terminally ill study population unable to be interviewed [49]. This high attrition rate is attributed to participants becoming more unwell or passing away during the study. [49, 50]. As patients deteriorate, the ethics of imposing the researcher’s demands at such a sensitive time also needs to be considered. Proxy interviews were therefore selected as the next best option for exploring the patient experience.

Despite the inherent limitations, the views of proxies are considered to have inherent value [51], as they form a key part of the unit of care. The proxy utilized here was the primary caregiver of the deceased. The caregiver plays a pivotal role at the end-of-life as an essential part of the health care team, often at significant personal cost [21, 52], while off-setting wider economic cost [53, 54]. The patient’s achieving death at home is strongly influenced by his having a caregiver’s support [55, 56]. The caregiver therefore directly impacts the patient’s end-of-life experience.Teno [57] suggested that our response as health care professionals to the voices of patients and families is perhaps the best outcome measure of the quality of our care. This study gives voice to some family caregivers.

Evidence is lacking for an ideal time frame for after-death interviews [46–48, 58]. While interviewing soon after an incident is likely to yield more accurate results, the challenge lies in being sensitive to the needs of the grieving family and of interviewing before recall bias is an issue [47]. In this study, interviews were conducted 6–12 months after death in the hope that a culturally acceptable period of mourning would have elapsed yet recall would still be accurate. Some researchers suggest that an acceptable approach may be leaving it to the participant to say if the timing is right [47, 59]. In this study, only one participant declined because of the perceived emotional burden, lending support to the 6–12 month time frame chosen.

### The research sample

Using purposeful sampling, participants were obtained by referral from health professionals at palliative care/oncology centres in four geographical areas: Port of Spain, Sangre Grande, San Fernando and Scarborough, Tobago. There was no prior relationship between researcher and participants. Participants were included if they were over age eighteen, English speaking and had been the primary caregiver for a cancer patient in Trinidad and Tobago, deceased in the preceding 6–12 months.

### Data collection

Ethical approval was obtained from the Ethics Department of the University of the West Indies and from the relevant Regional Health Authorities. Participants were contacted by telephone by KCS who introduced herself as a general practitioner with an interest in community palliative care. Prospective participants were informed that the aim of the study was to identify the unmet needs of cancer patients in their last year of life.

**Fig. 1:**
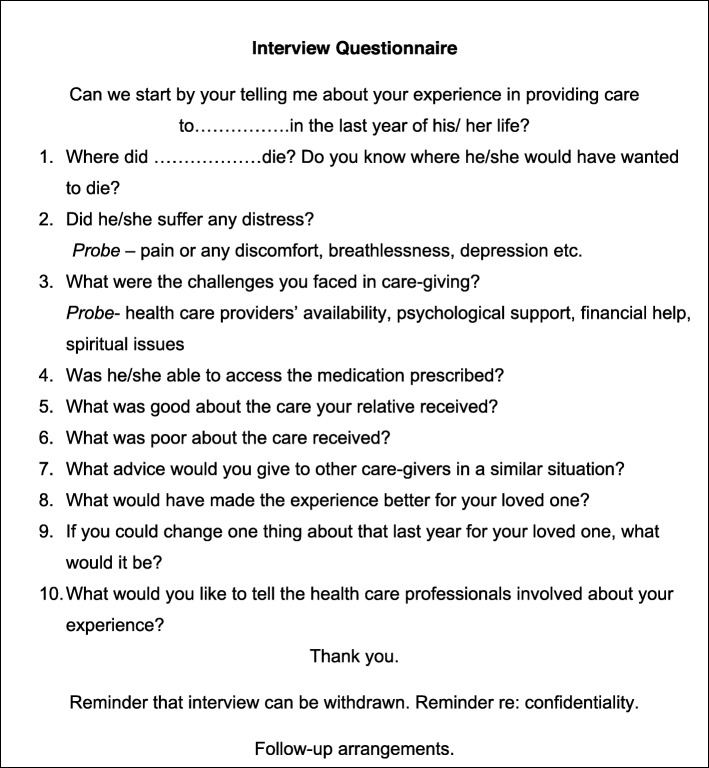
Interview Questionnaire

Written informed consent was obtained prior to interview. Interviews were conducted by KCS, with only participants and interviewer present. The semi-structured interview questionnaire (Fig. 1) was employed after being first pilot tested.

Interviews were audio-recorded with field notes made during and after interviews. Subject recruitment and interviewing were continued until data saturation was achieved. Data saturation was considered to be attained when additional data did not lead to the generation of additional perspectives [60].

### Data Analysis & Synthesis

Data collection and analysis took place concurrently. Thematic content analysis and was used to derive common themes. As both authors were of primary care backgrounds, a third coder and research assistant with a social science and education background was recruited, to lend diversity and a different perspective. Each transcript was coded by at least two researchers, with KCS being first reader on all. Prior to coding, the first author read and familiarized herself with the manuscript. Two coders then re-read the material, individually identifying and coding all narratives that spoke to the research question. Codes were then shared, discussed and the emerging common themes identified. Finally, a thematic map was proposed with the themes divided into major and minor ones. Analysis was iterative, with reflexivity a common thread throughout [61].

**Fig. 2:**
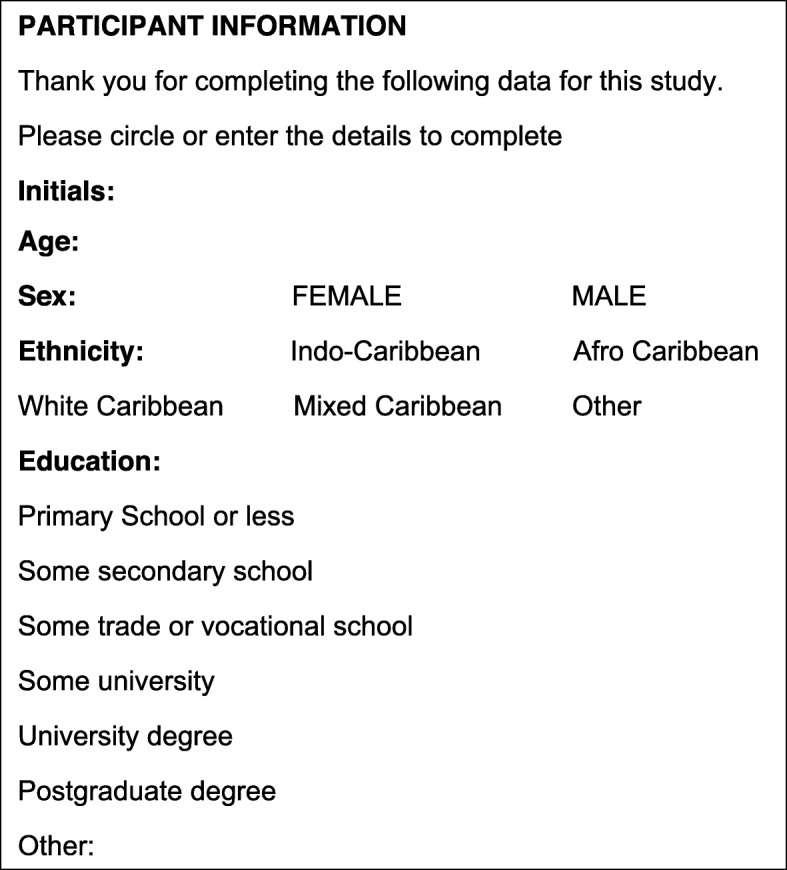
Data Collection Sheet for Participants

Identifiers were deleted, and transcripts stored separately from demographic data. Audio-recordings were destroyed after transcription. Electronic data was stored password protected, on a Cardiff University secure site. The data collection sheet utilized is shown in Fig. 2**.**

## Results

Of the forty-four possible interviewees referred, twenty-one met the eligibility criteria. Three of these were unavailable to meet for interview and three others declined: two citing reasons of ill-health and one stating that she was unwilling to relive the experience. Interviews were therefore conducted with fifteen [14] families - fourteen [13] from Trinidad and one [1] from Tobago. One repeat interview was required.

Interviewees ranged in age from twenty-nine [28] to seventy-seven [62]. Twelve [12] of the fifteen [14] participants were female. Eight [8] were children of the deceased, the others, spouses. Ten [10] interviews were done at the participant’s home; the others at medical institutions. Interviews lasted between one and two hours.

Ages of the deceased ranged from 43 to 93 with an average age of 65.5 years. Seven [7] were female. Seven [7] died at home, four [4] at a hospice and four [4] at hospital. See Tables 1 & 2 for participant and patient demographics.

**Table 1:** Participant Demographics Matrix

Participant Number	Age range	Ethnicity	Educational level attained	Location of Interview
001	60–64	AFR	PRIMARY	HOME
002	35–39	INDO	TRADE/VOCATIONAL	HOSPICE
003	35–39	MIXED	TERTIARY	HOSPICE
004	65–69	INDO	PRIMARY	HOME
005	55–59	INDO	SECONDARY	HOME
006	75–79	MIXED	TRADE/VOCATIONAL	HOME
007	50–54	MIXED	TERTIARY	OFFICE
008	45–49	MIXED	PRIMARY	HOME
009	65–69	AFR	TERTIARY	HOME
010	35–39	MIXED	TERTIARY	OFFICE
011	35–39	MIXED	PRIMARY	HOME
012	55–59	MIXED	SECONDARY	HOSPITAL
013	25–29	INDO	TERTIARY	HOME
014	55–59	INDO	SECONDARY	HOME
015	50–54	AFR	SECONDARY	HOME

**Table 2:** Patient Demographics Matrix

Patient Number	Age range	Religion	Site of Primary	Place of Death	Preferred place of death
001	50–54	R.C.	BREAST	HOME	HOME
002	50–54	R.C.	LUNG	HOSPICE	HOME
003	65–69	R.C.	PROSTATE	HOSPICE	HOME
004	90–94	HINDU	UNKNOWN	HOME	HOME
005	60–64	CHRISTIAN	OESOPHAGUS	HOME	HOME
006	80–84	R.C.	OESOPHAGUS	HOSPICE	NOT KNOWN
007	65–69	ANGLICAN	OESOPHAGUS	HOSPITAL	NOT KNOWN
008	45–49	HINDU	RECTAL	HOSPITAL	NOT KNOWN
009	90–94	R.C.	COLORECTAL	HOSPICE	HOME
010	40–44	R.C.	LUNG	HOME	HOME
011	60–64	R.C.	MAXILLARY ANTRUM	HOSPITAL	HOME
012	65–69	R.C.	PANCREAS	HOME	HOME
013	50–54	HINDU	LUNG	HOME	HOME
014	80–84	HINDU	OVARY	HOME	HOME
015	70–74	NOT KNOWN	BLADDER	HOSPITAL	NOT KNOWN

### Unmet needs

**Fig. 3:**
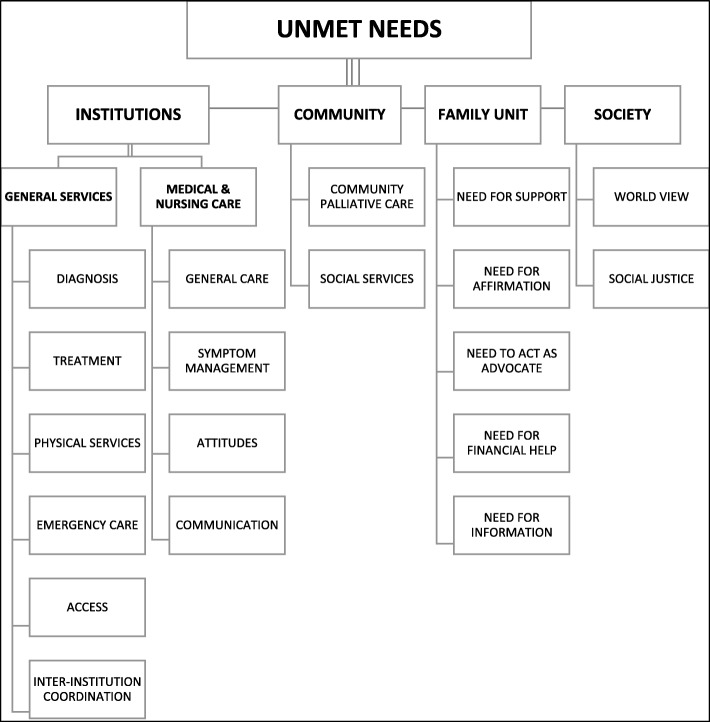
Major and Minor themes identified

While some positive outcomes were reported, unmet needs featured far from frequently. These unmet needs were classified into common themes under the following domains: Major theme: Institutions. Minor themes: Community, Family Unit and Society. These were further divided into several subthemes **(**Fig. 3**).**

## MAJOR THEME: INSTITUTIONS.

**SUBTHEMES: I. GENERAL SERVICES II. MEDICAL AND NURSING CARE.**

Criticisms of the public health institutions abounded. Particularly prominent were references to delays in the diagnostic step.

I. **GENERAL SERVICES**

**Subthemes**: a) Diagnosis b) Treatment c) Physical Facilities d) Emergency Care,

e) Access f) Inter-institutional coordination.

- **Diagnosis -** *“It take so long!” (F8)*

Time to diagnosis ranged from 1 month to more than 1 year. For six [6] patients it took over six [6] months to be diagnosed. The longest diagnostic delays were seen for patients with cancers of the oesophagus (2 patients), maxillary antrum, breast and lung. Participants described public healthcare system failures leading to diagnostic delays and poorer outcomes. Long waiting times for appointments and procedures were experienced, as well as multiple cancellations. Lack of proper communication and poor follow-up arrangements also contributed.

For these two widows whose husbands were diagnosed with oesophageal and colorectal carcinomas respectively, time to diagnosis was in the region of 1 year:

*I am saying that when your loved one is not feeling well and you see their eyes yellowing and stuff like that, you need answers… you need to feel like something is being done at a fairly decent pace, I think, and am… that wasn’t happening. (E9)*

*It was more than a year he do tests and tests and tests and they couldn’t tell him anything. Until almost the last… and it take so long for him to do that CT, till that time he started to… you know, get small... Oh boy, when we do CT scan… weeks, sometimes months before you could get a report! Until when they did the colonoscopy. Well he went about three, four times to do the colonoscopy. Sometime… once or twice the machine wasn’t working, another time his blood count was too low, they couldn’t do it. (F8)*

b) **Treatment -** *“the machine broke down” (B130)*

Having been diagnosed, caregivers reported many challenges faced by patients in getting timely treatment due to equipment failures and inadequate supplies. Chemotherapy and radiotherapy regimes had to be interrupted and operations re-scheduled. The daughter of a patient with lung cancer, who had already waited 6 months before being diagnosed, recalled:

*When she now started the radiation, in December, she got I think two treatments and she had to stop because the machine broke down, and we had to wait till January for them to fix it. (B130)*

For a patient with advanced rectal cancer, the delay was in having a nephrostomy tube inserted.

*Then the doctor come and say how the machine to put back in the tube to his back...is not working and the one that they has in theatre, they using it. So they give us another appointment, to come back the following Thursday. (H133)*

Caregivers described more timely access at private hospitals however this was at significant cost. This son, caring for his elderly father, sought medical attention first at the public hospital but the long waits and unpleasant conditions eventually led him to seek private health care. He described the private health system as mercenary.

*…now we’ve reached into a money world! We will do nothing until we get our money! (I50)*

*Fourteen hundred dollars (laughing)… I mean I shouldn’t say this, but what I feel is that…. (long pause)…once they realize that ok there’s nothing more can be done for you, let us see how much money we can take from you! (Laugh) (I218).*

c) **Physical Facilities –** *“if you are not sick, you will feel sick!” (I74)*

Participants decried the physical appearance of some public health institutions.

*First thing is the cleanliness…the appearance. First thing that hits you is the appearance of the surroundings. You go to Casualty, if you are not sick, you will feel sick! (I74)*

d) **Emergency Care** – *“a hopeless waiting scenario” (I56)*

Emergency care was a frequently cited area of unmet need. While some public Oncology services provided telephone contacts for daytime advice, out of hours cover was generally not available. After hours patients had to seek care at the nearest emergency department. Caregivers recounted the agony of waiting for up to fifteen [14] hours, some leaving because no care was forthcoming. This widow recalls waiting with her husband who had been transferred from another hospital but still needed to go through the emergency room process:

*You just waiting and waiting (pause) and waiting and…like the whole day just going and every time you go and ask, they say, yes, just now the doctor will see you. (J22)*

This son described visits to the emergency room during his father’s illness:

*I have had occasions to sit with him in the front part of casualty, in a kind of a hopeless waiting scenario. And it is undignified. It’s not… for a human being to be treated like that… (I56).*

e) **Access** *- “I mean, you driving from Toco, to spend one hour!” (D53)*

Public hospitals’ visiting hours were limited to two fixed periods per day. Families, especially from distant rural communities, struggled with the restricted visiting and unaccommodating staff attitudes. One widow who lived more than 2 hours’ drive from the general hospital, recounted the challenge of spending time with her blind husband.

*…they are not doing anything for him. Good? So if you have to go down to Port of Spain, you going to spend the whole day travelling, to get there for 4 o’clock, to be put out at 5 o’clock. (G63)*

Physical access to institutions was cited as an issue by some caregivers. Government Emergency Health Service (EHS) ambulances, although free, only transport patients to specific regional public hospitals. Families sometimes had to struggle with patients in cars or to pay private ambulances. On arrival at hospitals, they were faced with inadequate parking, uncooperative attendants and lack of designated drop-off bays.

This daughter described the process of transporting her mother to hospice for admission:

*…it was torture for her ‘because she had to lie down in the back (of the car) and as I said she was heavy set and even when we reach here, I had to take her out. The nurses couldn't and they were like, "Yuh didn't bring a man with you?" (B88)*

Another caregiver described the challenge of dropping her husband off for treatment at the Radiotherapy Centre.

*…even to drop him off…obviously you have to stop to go to call the attendant… and the guards are just yelling at you, “You can’t park there! You can’t park there!” Eventually I would just say, “Move the car!” Good! (G84)*

f) **Inter-institutional Co-ordination -** ***“****they just wanted to do their own thing” (D47)*

Caregivers reported that patients referred from one facility to another often found themselves at the mercy of the host institution. Direct admissions were not possible, resulting in patients having to endure the tedious emergency room process.

*He saw the doctor and everything…but he say in order to ward him, to go upstairs, he had to go through the process. My husband can’t stand up, he can’t walk, he can’t do anything…because I mean, is a patient who came from the hospital and can’t sit down, can’t do anything more than lie down… that is all he was doing when he was in the hospital, lying down, and you telling me he had to go over that whole process…Doesn’t make sense. (J20)*

Another recounted the emergency room team choosing to institute a different plan from the one requested.

*He only came in to get an ECG done to see whether they could continue that week with the radiation… and you know, as if they don’t listen to what the patient is saying. The caregiver or the patient is saying…. they just wanted to do their own thing. (D47)*

On the smaller island of Tobago, this caregiver reported even greater logistical challenges in accessing specialist medical care. Poor coordination and communication between institutions in Tobago and Trinidad resulted in several futile inter-island trips:

*They had nobody to do it over here…sometimes she got down (to Trinidad) and machine was down… there were times when we left here and went down there and could not do anything and we had to come back…We went … a couple times. More than six… (O7)*

II. **MEDICAL & NURSING CARE –**

*“She wasn’t getting the care and attention there, trust me.”* (A57)

Subthemes: a) General Care b) Symptom Management.

c) Attitudes d) Communication.

- **General Care** *– “I think no one took the time” (E15).*

Participants described inattentiveness and insensitivity of staff. Lack of attention to the feeding of patient featured prominently.

*… I think they should have something in place…like just to feed who can’t help themselves, to… give them that attention. (J15)*

*I think no one took the time. Maybe because they didn’t have the time… they would bring the water and they would put it down there. Sometimes I feel that am it wasn’t even within his reach…sometimes. (E15)*

Some were so concerned about the care that they were afraid to leave their loved ones.

*And then this is when you start to get nervous now about leaving them alone, because you wonder… (G39)*

b) **Symptom Management** -“*Bawling with pain” (B27)*

Pain was the most commonly mentioned symptom, with all but two of the narratives describing uncontrolled pain.

Caregivers described health professionals who seemed reluctant to prescribe opioids, delayed administration until late and utilized ineffective dosages. In all the cases where patients suffered pain, access to analgesia, often Morphine, was reported. This was obtained free of charge from government pharmacies.

*She whole body was pain… (plays a recording from his phone of a woman screaming in distress)…Yeah, day and night, day and night… (K158)*

*…they will wait till she can’t bear it anymore and then gave her something. (M222)*

*…we did not know that the pain was going to be so bad that there was nothing in this world that we could try that will help it… (N233)*

Poor symptom management resulted in multiple admissions. Many caregivers reported more than three admissions in the last year of life; others had lost count, simply recalling being constantly in and out of hospital.

c) **Attitudes** – *“Very cold and very uncaring” (D19)*

While there were descriptions of laudable staff attitudes, notably in staff from oncology, palliative care and the Sangre Grande District Hospital, most caregivers expressed dissatisfaction with the care received at government hospitals. These caregivers described a lack of empathy, rigidly enforced hospital regulations and insensitivity to patient need.

*…because they always say part of the process, the whole medical healing process, is the family involvement. But at Hospital Y, you find that they block you out…I went into Casualty with him, the first day I said, “visually impaired”, so I would need to be there. The guard let me in. The first set of nurses were fine. While we were waiting, change of shift at 3o’clock, “Well you will have to go outside!” I said, “But he can’t see, you know!” (G34)*

*… she walk in and she say, “Well here is for patients only, you know! You cannot stay here.” My husband sick, he can’t stand up he can’t sit down, he can’t… “No, yuh cannot be here, you have to move from here!” (J28)*

d) **Communication -** *“they wasn’t telling us nothing” (B84)*

Many of those interviewed described wanting more information and having difficulty gaining access to doctors to obtain it. These widows of different generations and educational backgrounds, the first elderly and poorly educated and the second, middle-aged and well-educated, described similar issues:

*Ah never… I never saw a doctor, because when we go the doctors not around. We just see the nurses and ask how he’s doing and they say well he doing ok…We didn’t really get information. (Laugh) We didn’t really get information. (F48)*

*Then the doctors are there at 7 o’clock or at 9 o’clock, when visitors are not around, so that means we, the family, doesn’t get to interact with the doctors and ask questions. (G57)*

This daughter found the doctors reluctant to discuss her father’s prognosis.

*I’d get all these vague, indecisive…and I’d tell them listen, I don’t need to be sugarcoated… I need to know, what is the prognosis? (C60)*

*I wanted to take the leave of absence but because I did not have a straight answer to know how long it would be… so I have one doctor telling me it might be two years; I have one doctor telling me they don’t know. (C38)*

When conversations did occur, communication skills were deficient, as one participant, who had attained education only up to a primary school level, explained:

*… my daughter now used…to communicate with the doctor because most of the time I couldn’t understand what dem saying much. (A67)*

Some participants lamented that bad news was given in a manner that robbed them of hope.

*He took away her hope…Go home and sit down and wait till you die (N113)*

Goals of care discussions were lacking. The wife of a young cancer patient described standing helplessly by, unable to meet with the doctors as her husband was put through what she recognized as futile treatment.

*I even went and I tell the nurse…I say…he have three more treatments (radiotherapy)…I say it doesn’t make sense to carry him, you know? I ask she, I say, not to carry him. She say I have to ask the doctor. I sit down and I wait. I didn’t see the doctor. When I left, the next day, they carry him again and they do the treatment again. (J59)*

Another caregiver reported her father taking treatment cessation decisions into his own hands.

*…because he had to say, this is not working, I am not doing this anymore…my body can’t take any more chemo. You can’t be putting me on chemo after chemo after chemo when it’s not working. I need to come off. I need to stop! (C124)*

## MINOR THEME: COMMUNITY.

**Subthemes:** a) Community Palliative Care b) Social Service programmes

- **Community Palliative Care**

Most caregivers reported that the patient’s wish had been to die at home but only about half achieved this. This young widow described her husband’s reaction to being told he would be taken home. He had spent many of the 20 months between onset of illness and death in and out of medical institutions.

*When I told him, I say, I carrying you home, I carrying you home… I say, because they can’t do anything for you again. And you could see the…you know you could smile with your eyes? You could see…and he smile. He actually smile. And I say, yes, that is what he wanted. (J104)*

Home care was challenging without community care infrastructure. Caregivers described concerns around meeting nutrition and hydration needs and managing oxygen and colostomies. They described helplessness and despair.

. *I wasn’t equipped to do anything for him. (F37)*

*There were times when we would be so helpless and I think, you know, knowing that she had like a nurse or doctor somebody nearby to help her that would have been good. (N250)*

In the limited situations where community palliative care or hospice admission was available, caregivers reported tremendous relief and comfort as the burden of care was lifted.

b) **Social Service Programmes** - *“that didn’t come through…” (B129)*

The Geriatric Adolescent Partnership Programme (GAPP) is a governmental initiative which provides caregivers for the elderly in the community. For many of the caregivers surveyed, the delay in initiation of this service rendered it inaccessible.

*I think it is called GAPP… I went on the wrong day apparently, because they only do the interviews on the Wednesday, but they don’t tell you that over the phone… I called for about two months….the lady who does the interviews, has to do the site visit… I kept calling her to ask… if she could send somebody…she kept saying she’d try and we went on and on and on, until I stopped calling. (C110)*

*We had applied for nurses through the government...But that didn’t come through. That didn’t come through. (B129)*

Similar red tape was described as caregivers tried to access other government social services. Grants remained unapproved even after the applicant’s death.

*… I think the Community Development does give you a wheelchair, but you know you have so much a forms to fill out and so much a things to go through…before you could get a chair…and you have to go to Port of Spain, you know? They wouldn’t make thing easy, like in an office. (D180)*

Social worker input and counselling were rarely available to the caregivers interviewed. Families were uninformed about where they could access help. Resource centres that should have been meeting this need were inaccessible.

*I think people need to have somewhere they can go to... once your patient is diagnosed… because you yourself frightened. I was frightened…but I started to look for information. I remember being so afraid. (E16).*

*… you only hear about the Cancer Center X but I don’t know if they even active because I try calling numerous times. Nobody answered. There is a machine coming on and I left the contact information. They never called … is like… you in a desperate attempt to just try to get someone to talk… to, like gain a little positive… a little hope I think. (N151).*

## MINOR THEME: THE FAMILY UNIT.

**Subthemes:** a) Need for support b) Need for affirmation c) Need to act as advocate d) Need for financial help e) Need for information.

This study aimed to investigate the needs of the patient through the eyes of the caregivers, but the caregivers frequently recounted issues that they themselves faced, which impacted the patient experience. Some of these are therefore reported here.

- **Need for Support**

*“Probably I tried to be superwoman” (B26)*

Caregivers reported their struggles to care for the patient, with their own needs going largely unrecognized. These needs ranged from physical help with caregiving to needing affirmation to assuage guil**t.** The struggle to balance complex care needs often came at great personal cost.

This participant struggled with caring for a toddler and an ill husband. The additional pressure of in-laws blaming her for her husband’s illness led her to despair.

*I was at a stage where I didn’t know what to do, and I say, what to do, just kill myself and my child and leave them? (J15)*

The only daughter of this patient described how difficult it was it to multitask and still be attentive to her father’s emotional needs.

*I had to do the driving and do the pick-up and deal with the money and be sensitive and be caring and make sure you had this and that and the other. A couple times he said, you know, you could be a little more sensitive, and I said, I don’t have time to be sensitive. (C44)*

b) **Need for Affirmation**

Guilt was commonly expressed. Caregivers needed reassurance that they had done enough.

*Sometimes I does feel like, like…like… is my fault…that he died before time. I doesn’t know how to think, because sometime, because sometime I think if he did go there (private hospital), he’d of get a different treatment. (J88)*

*…it’s funny that I did all these things and I still feel guilt… I still think back, oh gosh! You could’ve been a little more patient…once or twice I came home and I saw him crying and I’d be like (patting self on shoulder), “OK, alright, alright, I’m coming back just now.” You know, because I am exhausted and I’m tired and I know you’re crying. I’ll come when I have a shower, or get something to eat… I’m going to come back… (C42)*

c) **Need to act as Advocate**

In the face of systemic inefficiencies, caregivers were forced to act as act as patient advocates to ensure that the patient’s needs were met.

*“They don’t have any more stents.” So I say, “Can I see the doctor? The nurse? I need to find out what is the next step!” Good! Well I had to stay there about 7 or 8 o’clock the night to see the doctor…but I was determined not to go… I had to say, “All yuh must get the stents from somebody. Who is the supplier? I will buy the stent!” And this is how he got his stent! Good? (G31)*

*… you must have somebody with you, when you are a cancer patient…who cares. For you to survive! (H28)*

d) **Need for Financial Help**

Caregivers described the economic burden of ongoing care. To ensure that patients got the best possible care, families held fund-raising events, took loans and generally struggled to meet expenses.

*… I suppose they expect you to mortgage your house, so when the patient dies, you have nothing. (G182)*

*… we didn’t even had the money to bury her. Is my boss and them came and try to help… (K97)*

e) **Need for Information**

Caregivers reported needing accessible, practical information to empower them in caregiving.

*I felt… that he was sent home to die. It didn’t matter how much I know, to take how care of him. (E139)*

*I tried to do like online research…but I really didn’t have the time to sit down and filter through all of these websites to find exactly what I needed. (C59)*

*There is no real information or system for the community, let’s say if I need a wheelchair, to know what to do. (E61)*

## MINOR THEME: SOCIETY.

**Subthemes:** a) Worldview b) Need for social justice.

- **Worldview** - **“***somebody do him something” (J6).*

The narratives revealed culturally ingrained belief systems around alternative medical practices and beliefs in obeah/witchcraft. These beliefs were noted to be held by participants of lower educational levels. Illness was believed to be cast upon individuals by the envious. Causes ascribed to ill health were trauma, interventions such as surgery and stressors such as family conflict.

*Yeah, mix your bush, herbs. Drink your herbs and try and clear out whatever sickness you have. (K56)*

*So I started to think otherwise...(lowered voice), like… somebody maybe hurt her…It have wicked people out there you know! (A8)*

*They blamed me because they thought maybe somebody do him something…and they thought it was me…well they believe like…witchcraft or something like that. (J6)*

Misconceptions and stigma around hospice care and morphine use were revealed. These misconceptions seemed to be quite widely held, expressed by participants of varying educational backgrounds. Hospice was viewed as a place of no return and Morphine as an addictive substance, to be reserved for extreme pain or the very end.

*I didn’t want to bring him here. I had a perception of what a hospice was… everybody had a perception of what a hospice was. (C171)*

*We realized, well…you really don’t come out of there too much. (I159)*

*Knowing morphine was addictive drug, she didn’t want that. (E247)*

*He just said well, he didn’t think he had enough pain for that… knowing it was Morphine. (G210)*

*Because I feel morphine is so final. (E75)*

An unexpected finding reported by caregivers was that some staff at public institutions held a very a negative attitude towards cancer patients.

*You know he’s stage four, so he’s going to die and full stop. So…he’s just waiting. And that wasn’t nice. (H44)*

*I had two people just looked at me like… he’s just another patient. He dying, he will die. And I’m talking about doctors. (G43)*

*…but when the registrar came, she stands at the foot of the bed and says, “Well you know he’s a cancer patient.” So in other words, because he’s a cancer patient, c’est la vie. (G98)*

b) **Need for Social Justice** - *“money talks and bullshit walks” (B54)*

Participants lamented the unfairness of the social order – the ineligibility of law- abiding citizens for government funding to assist with care and the discrimination towards the economically disadvantaged.

This widow expressed anger at being excluded from financial assistance.

*I find certain basic things should just kick in, across the board…because during that time I did a lot of reading on the social services and I can tell you, it pays not to go to school. Right! And GAPP, as long as you make more than $5000 a month, you’re not eligible for it. And woe betide if you have worked for all these years as a civil servant. (G259)*

The economically disadvantaged son of another patient described feeling marginalized in accessing health care due to a lack of money and connections.

*I realizing… is like, when you go into the hospital, and you real sick and thing, is who know you and who you know, for instance you go in the hospital, if one of the doctors there know you very good, and you know him, probably you might get a better treatment, you understand? I watch it that way….And most of the time too like out here too, like money talks and bullshit walks. Yeah. (B54)*

## Discussion

This study demonstrated the range of unmet needs experienced by cancer patients in T&T as reported by their families and caregivers.

### Delay in diagnosis and treatment

Widespread system failures led to delays in the public hospitals’ processes. An overburdened service accounts for some of these delays, but in some cases, participants held the physician responsible. In a country where a large proportion of cancer patients receiving treatment are non-curative diagnostic delay is likely a significant contributor to late stage disease. Trinidad and Tobago needs to focus on improving its facilities, equipment maintenance and institutional processes in all public health care settings. Instituting measures to minimize this delay could be crucial to changing the country’s cancer landscape and improving outcomes.

### Pain management

Pain was poorly controlled in this population. Pain is experienced by 70–100% of palliative patients [63], and pain management is a challenge in developed countries [64] as it is in the Caribbean [65]. Several barriers to effective pain control were reported in this study, but interestingly, these did not include unavailability of analgesics.

In many Latin American and Caribbean territories, including Trinidad and Tobago, opioid consumption is low compared to the global mean [66]. Disproportionately high cost relative to income is a barrier in some countries [67, 68], while stringent regulatory restrictions operate in others [69]. In terms of restrictive policies for opioid prescribing, Trinidad and Tobago ranks low [66].

All those reporting pain in this study had access to analgesics, often morphine, free from government pharmacies, so pain went unrelieved despite morphine availability. This contrasted with the Jamaican finding that over two-thirds were unable to purchase medication [45]. In Grenada, drug shortages contributed to poor pain management, but other factors identified were reluctance to use medication and ineffectiveness of drugs prescribed [44]. The latter two factors mirrored this study’s findings, with stigma around Morphine use and ineffectiveness of prescribed doses.

The narratives suggested physician reluctance to prescribe strong opioids and reluctance of health workers to administer Morphine until pain was severe. These findings speak to the need for targeted health professional education on cancer pain management.

### Communication and attitudes

Inadequate communication was cited by about half the participants as a major unmet need. In studies of end-of-life care, this is consistently ranked as a critical need [13, 14, 24, 38]. Poor communication was distressing to caregivers in this study. As seen in other work, this lack of consistent communication can contribute to an increased burden of care [36] and to adverse bereavement experiences [38].

The “information giving behaviours” which foster effective communication [70] were found lacking. This theme was also evident in Jamaica, where inaccessible language and a reluctance of physicians to tell patients the truth, were reported [45]. In this study, both communication skills and opportunities for information sharing were deficient. Health professionals’ inadequacy in communicating may have contributed to their avoiding difficult conversations. Limited visiting hours contributed, denying families access to wards when doctors were around.

This reluctance to share information suggests a failure to recognize the pivotal role of the caregiver as part of the unit of care and the wider health system [21, 71, 72]. Families need to be valued for their critical role in caregiving. Community based interventions built on lay persons have been highly effective in places such as Kerala [73, 74].Trinidad and Tobago might benefit from an approach that focuses on valuing the caregiver, putting interventions in place to train and educate the caregiver [36, 37] and on harnessing community resources to improve quality of care.

### Social services

The GAPP programme has tremendous potential to meet the needs of patients in the community, but the long lag time for initiation of services failed most of these patients with short prognoses. The bureaucracy around applying for other social service benefits was similarly a deterrent.

### Needs of the family unit

This study confirmed the findings of many others internationally, that care-givers need support [19, 37, 62, 75, 76], often ignore their own needs [20] and bear the burden of advocacy [13, 14] and guilt [38, 77]. The family unit needs information [26, 31, 35, 36, 78, 79] and financial help [62, 80, 81].

The economic burden of caregiving was borne by participants, who needed to hold fund-raising events and take loans to cover care costs. A social service benefit akin to that of the UK’s Attendance Allowance, payable to persons in need of care, and expedited for those with prognoses of less than six [6] months, could be a consideration.

As reported in Jamaica [45],caregivers felt ill-equipped to care for loved ones at home. Many voiced distress at being alone. Where palliative care teams/hospice care existed, the distress was alleviated, but these services were very limited. Establishment of an interconnecting network of palliative care services, in each Regional Heath Authority, could help meet this need.

Lack of support impacts the physical and mental well-being of the caregiver [62, 77, 81, 82] and can lead to the care-giver himself becoming in need of care [82–86]. This jeopardizes the care of the patient, adding to an already over-burdened health system. The needs of caregivers need to be recognized and addressed.

### Worldview

The narratives revealed cultural illness beliefs with supernatural and spiritual undertones. Spiritual meanings were attached to disease processes that seemed inexplicable, such as disfigurement and extreme pain. There was delay in seeking care in two instances because the tumour was attributed to trauma. Some believed that the disease had been cast upon the patient by the envious, using witchcraft or obeah. Similar beliefs were described by Spence et al. in Jamaica where cancer was viewed as a curse or retribution from God and obeah invoked as a cure [45]. The findings align with those in indigenous communities, where supernatural, natural and societal contributions to ill-health are recognized [87].

In the Caribbean, “magico-religious” belief systems are observed to have persisted despite the emergence of bio-medical models of illness understanding [88]. These beliefs are believed to be an inheritance from West African forefathers. Studies in other patient populations have reported similar supernatural illness beliefs as more prevalent among black Caribbean people than their white British counterparts [89, 90].The caregivers disclosing these beliefs in this study were of Afro-Caribbean and mixed-Afro-Caribbean ethnicity and of lower educational backgrounds (completed education up to primary school level only).

Myths about morphine and misconceptions about hospice affected drug compliance and uptake of services. Opiophobia is a major barrier to effective pain control not restricted to resource-poor settings, but described worldwide [91–96]. Opiophobia among lay persons as well as health professionals must be addressed if cancer pain is to be effectively managed. There is evidence in resource-poor settings such as Vietnam and Uganda, for the success of strategies that involve engaging governmental support at the policy level and developing education programmes that are culturally relevant [11, 93].

The finding of discriminatory and fatalistic attitudes towards cancer patients by hospital staff, including physicians was troubling. Spence [45] reported stigmatization of cancer patients in Jamaica resulting in neglect and abuse of the patient by family. Cancer fatalism among lay-persons has been recognized as a barrier to health-seeking behavior [97], but data on cancer fatalism among health professionals is lacking. An ethnographic study in India described fatalistic attitudes among health professionals in dealing with cancer pain, which they felt was “inescapable and unmanageable” [98]. Perhaps the health professional’s feeling of powerlessness in the face of incurable disease and distressing symptoms engenders these attitudes. The fatalism described among some Trinidadian health professionals warrants further exploration.

### Limitations

This small, retrospective study may have some inherent limitations.

One limitation relates to the use of proxies.The views of proxies and patients have been found to have a high degree of concordance for objective issues and those around service provision but are less reliable for subjective domains such as pain and psychological symptoms [99, 100]. Family caregivers have been shown to report more negatively on quality of life issues than patients [101, 102]. These limitations must be borne in mind in interpreting results.

Referral of participants by health professionals may have biased results. Williams, Woodby [47] noted that an individual’s continuing relationship with an institution can influence participation in research of that institution.

Referred participants might report more favourably than those randomly selected. The fact that the interviewer was not known to the participants or affiliated with the organizations from which participants were referred, should have helped to minimize this.

Sampling only one family from Tobago was another limitation. The Tobago end of life experience is likely to differ from that on the larger island of Trinidad, but with only one experience captured, this could not be adequately explored.

Analyst triangulation was utilized in the collaboration of three researchers, at least two of whom analyzed each transcript. Peer review was used to ensure accurate representation of participants’ views. Respondent validation or member checks were attempted, albeit not very successfully. Seven participants were contacted, one did not respond and another cited time-constraints. Two reported distress on re-reading the transcripts. The burden placed on participants by this process was acknowledged and further member checks not pursued.

## Conclusions

The purpose of this report was to describe the unmet needs identified by families and caregivers of persons who recently died from cancer in T&T. Many of the identified themes of unmet need resembled those seen in the global literature.

Recommendations to improve end-of-life care in Trinidad and Tobago must address issues at the national, institutional and societal levels.

## Data Availability

The transcribed interviews are available on reasonable request from the corresponding author, KCS.

## Abbreviations

AFR: Afro-caribbean
E.H.S.: Emergency health services
INDO: Indo-caribbean
N/A: Not applicable
P.C.U.: Palliative care unit
R.C.: Roman catholic
R.H.A: Regional health authority

## References

[1] Gwyther L, Brennan F, Harding R (2009). Advancing palliative care as a human right. J Pain Symptom Manag.

[2] Brennan F (2007). Palliative care as an international human right. J Pain Symptom Manag.

[3] World Health Assembly (2014). Strengthening of palliative care as a component of comprehensive care throughout the life course.

[4] World Palliative Care Alliance and World Health Organization (2014). Global atlas of palliative care at the end of life.

[5] Webster R, Lacey J, Quine S (2007). Palliative care: a public health priority in developing countries. J Public Health Policy.

[6] World Health Organization (1996). Cancer pain relief with a guide to opioid availability.

[7] Basu A, Mittag-Leffler BN, Miller K (2013). Palliative care in low- and medium-resource countries. Cancer J.

[8] Pan American Health Organization (2013). Cancer mortality is declining in some countries of the Americas - - new PAHO/WHO report.

[9] National strategic plan for the prevention and control of non communicable diseases: Trinidad and Tobago 2017-2021. 2017. http://www.health.gov.tt/ncd/. Accessed 17 Jan 2020.

[10] Shetty P (2010). The parlous state of palliative care in the developing world. Lancet.

[11] Clark D, Wright M, Hunt J, Lynch T (2007). Hospice and palliative care development in Africa: a multi-method review of services and experiences. J Pain Symptom Manag.

[12] Pastrana T, Eisenchlas J, Centeno C, De Lima L (2013). Status of palliative care in Latin America: looking through the Latin American atlas of palliative care. Curr Opin Support Palliat Care.

[13] Teno JM, Casey VA, Welch LC, Edgman-Levitan S (2001). Patient-focused, family-centered end-of-life medical care: views of the guidelines and bereaved family members. J Pain Symptom Manag.

[14] Stajduhar KI, Funk L, Cohen SR, Williams A, Bidgood D, Allan D (2011). Bereaved family members assessments of the quality of end-of-life care: what is important?. J Palliat Care.

[15] Gallagher R, Krawczyk M (2013). Family members' perceptions of end-of-life care across diverse locations of care. BMC Palliat Care.

[16] Howell Doris, Brazil Kevin (2005). Reaching Common Ground: A Patient-Family-Based Conceptual Framework of Quality EOL Care. Journal of Palliative Care.

[17] Dumitrescu Luminita, Van Den Heuvel Wim (2007). Evaluation of Palliative Care at Home: The Families’ Perspective. Journal of Palliative Care.

[18] McLaughlin D, Sullivan K, Hasson F (2007). Hospice at home service: the carer’s perspective. Support Care Cancer.

[19] Brazil K, Bainbridge D, Ploeg J, Krueger P, Taniguchi A, Marshall D (2012). Family caregiver views on patient-centred care at the end of life. Scand J Caring Sci.

[20] Fisker T, Strandmark M (2007). Experiences of surviving spouse of terminally ill spouse: a phenomenological study of an altruistic perspective. Scand J Caring Sci.

[21] Funk L, Stajduhar K, Toye C, Aoun S, Grande G, Todd C (2010). Part 2: home-based family caregiving at the end of life: a comprehensive review of published qualitative research (1998-2008). Palliat Med.

[22] Pazes Maria, Nunes Lucília, Barbosa António (2014). Factors influencing the experience of the terminal phase and the grieving process: the primary caregiver’s perspective. Revista de Enfermagem Referência.

[23] Phillips LR, Reed PG (2009). Into the abyss of someone else's dying: the voice of the end-of-life caregiver. Clin Nurs Res.

[24] Newbury J. The drama of end of life care at home. Nurs Times. 2011;107(11):20–3. 21682166

[25] Ventura AD, Burney S, Brooker J, Fletcher J, Ricciardelli L (2014). Home-based palliative care: a systematic literature review of the self-reported unmet needs of patients and carers. Palliat Med.

[26] Rhodes RL, Mitchell SL, Miller SC, Connor SR, Teno JM (2008). Bereaved family members' evaluation of hospice care: what factors influence overall satisfaction with services?. J Pain Symp Manage.

[27] Kristjanson LJ (1989). Quality of terminal care: salient indicators identified by families. J Palliat Care.

[28] Lecouturier Jan, Jacoby Ann, Bradshaw Colin, Lovel Tim, Eccles Martin (1999). Lay carers' satisfaction with community palliative care: results of a postal survey. Palliative Medicine.

[29] Keegan O, McGee H, Hogan M, Kunin H, O'Brien S, O'Siorain L (2001). Relatives' views of health care in the last year of life. Int J Palliat Nurs.

[30] Milberg Anna, Strang Peter, Carlsson Maria, Börjesson Susanne (2003). Advanced Palliative Home Care: Next-of-Kin's Perspective. Journal of Palliative Medicine.

[31] Addington-Hall JM, O’Callaghan AC (2009). A comparison of the quality of care provided to cancer patients in the UK in the last three months of life in in-patient hospices compared with hospitals, from the perspective of bereaved relatives: results from a survey using the VOICES questionnaire. Palliat Med.

[32] Heyland DK, Dodek P, Rocker G, Groll D, Gafni A, Pichora D (2006). What matters most in end-of-life care: perceptions of seriously ill patients and their family members. CMAJ.

[33] Beccaro M, Caraceni A, Costantini M (2010). End-of-life care in Italian hospitals: quality of and satisfaction with care from the caregivers' point of view - results from the Italian survey of the dying of cancer. J Pain Symptom Manag.

[34] Heyland D. K., Cook D. J., Rocker G. M., Dodek P. M., Kutsogiannis D. J., Skrobik Y., Jiang X., Day A. G., Cohen S. R. (2010). Defining priorities for improving end-of-life care in Canada. Canadian Medical Association Journal.

[35] Mayland CR, Mulholland H, Gambles M, Ellershaw J, Stewart K (2017). How well do we currently care for our dying patients in acute hospitals: the views of the bereaved relatives?. BMJ Support Palliat Care.

[36] Ateş G, Ebenau AF, Busa C, Csikos Á, Hasselaar J, Jaspers B (2018). “Never at ease” – family carers within integrated palliative care: a multinational, mixed method study. BMC Palliat Care.

[37] Harrop E, Byrne A, Nelson A (2014). “It’s alright to ask for help”: findings from a qualitative study exploring the information and support needs of family carers at the end of life. BMC Palliat Care.

[38] Harrop E, Morgan F, Byrne A, Nelson A. “It still haunts me whether we did the right thing”: a qualitative analysis of free text survey data on the bereavement experiences and support needs of family caregivers. BMC Palliat Care. 2016;15(1):–92. 10.1186/s12904-016-0165-9 PMC5101847 27825330

[39] Odgers J (2018). No one said he was dying: families' experiences of end-of-life care in an acute setting. Aust J Adv Nurs.

[40] Maharaj S, Harding R (2016). The needs, models of care, interventions and outcomes of palliative care in the Caribbean: a systematic review of the evidence. BMC Palliat Care..

[41] Hannon B, Zimmermann C, Knaul FM, Powell RA, Mwangi-Powell FN, Rodin G (2016). Provision of palliative care in low- and middle-income countries: overcoming obstacles for effective treatment delivery. J Clin Oncol.

[42] Silbermann M, Fink RM, Min SJ, Mancuso MP, Brant J, Hajjar R (2015). Evaluating palliative care needs in middle eastern countries. J Palliat Med.

[43] Rajagopal MR, Venkateswaran C (2003). Palliative care in India: successes and limitations. J Pain Palliat Care Pharmacother.

[44] Kreitzschitz K, Macpherson CC (2003). End of life care. Perspectives from families and caregivers. West Indian Med J..

[45] Spence D, Crath R, Hibbert A, Phillips-Jackson K, Barillas A, Castagnier T (2010). Supporting cancer patients in Jamaica--a needs assessment survey. West Indian Med J.

[46] Saunders D, Twycross R (2000). Why are trials in palliative care so difficult?. Palliat Med.

[47] Williams BR, Woodby LL, Bailey FA, Burgio KL (2008). Identifying and responding to ethical and methodological issues in after-death interviews with next-of-kin. Death Stud.

[48] Fowler F, Coppola K, Teno J (1999). Methodological challenges for measuring quality of care at the end of life. J Pain Symp Manage.

[49] Addington-Hall JM, MacDonald LD, Anderson HR, Chamberlain J, Freeling P, Bland JM (1992). Randomised controlled trial of effects of coordinating care for terminally ill cancer patients. BMJ..

[50] McWhinney IR, Bass MJ, Donner A (1994). Evaluation of a palliative care service: problems and pitfalls. BMJ..

[51] Addington-Hall J, McPherson C (2001). After-death interviews with surrogates/bereaved family members: some issues of validity. J Pain Symp Manage..

[52] Ornstein KA, Kelley AS, Bollens-Lund E, Wolff JL (2017). A national profile of end-of-life caregiving in the United States. Health Aff (Millwood).

[53] Rhee Y, Degenholtz HB, Lo Sasso AT, Emanuel LL (2009). Estimating the quantity and economic value of family caregiving for community-dwelling older persons in the last year of life. J Am Geriatr Soc.

[54] Grande G, Stajduhar K, Aoun S, Toye C, Funk L, Addington-Hall J (2009). Supporting lay carers in end of life care: current gaps and future priorities. Palliat Med.

[55] Wahid AS, Sayma M, Jamshaid S, Da K, Oyewole F, Saleh D (2018). barriers and facilitators influencing death at home: a meta-ethnography. Palliat Med.

[56] Gomes B, Higginson IJ (2006). Factors influencing death at home in terminally ill patients with cancer: systematic review. BMJ..

[57] Teno J (1999). Putting patient and family voice back into measuring quality of care for the dying. Hosp J.

[58] Thompson G, Chochinov H (2006). Methodological challenges in measuring quality care at the end of life in the long-term care environment. J Pain Symp Manage..

[59] Bentley B, O'Connor M (2015). Conducting research interviews with bereaved family carers: when do we ask?. J Palliat Med.

[60] Glaser BG, Strauss AL (1967). The discovery of grounded theory: strategies for qualitative research.

[61] Denzin NK, Lincoln YS (2005). SAGE handbook of qualitative research.

[62] Morasso G, Costantini M, di Leo S, Roma S, Miccinesi G, Merlo DF (2008). End-of-life care in Italy: personal experience of family caregivers. A content analysis of open questions from the Italian survey of the dying of Cancer (ISDOC). Psychooncology..

[63] McGuire DB (2004). Occurrence of cancer pain. JNCI Monographs.

[64] Teno JM, Freedman VA, Kasper JD, Gozalo P, Mor V (2015). Is care for the dying improving in the United States?. J Palliat Med.

[65] MacPherson C, Aarons D (2009). Overcoming barriers to pain relief in the caribbean. Dev World Bioeth.

[66] Cleary J, De Lima L, Eisenchlas J, Radbruch L, Torode J, Cherny NI (2013). Formulary availability and regulatory barriers to accessibility of opioids for cancer pain in Latin America and the Caribbean: a report from the Global Opioid Policy Initiative (GOPI). Ann Oncol.

[67] Wenk R, Bertolino M, De Lima L (2004). Opioid analgesics in Latin America: the barrier of accessibility is higher than the disponibility. Medicina Paliativa.

[68] De Lima L, Pastrana T, Radbruch L, Wenk R (2014). Cross-sectional pilot study to monitor the availability, dispensed prices, and affordability of opioids around the globe. J Pain Symptom Manage.

[69] Cherny N (2012). The international collaborative project to evaluate the availability and accessibility of opioids for the management of cancer pain: Survey result. Ann Oncol.

[70] Kristjanson LJ (1986). Indicators of quality of palliative care from a family perspective. J Palliat Care.

[71] Aoun S, Kristjanson L, Currow D, Hudson P (2005). Caregiving for the terminally ill: at what cost?. Palliat Med.

[72] Nakamura S, Kuzuya M, Funaki Y, Matsui W, Ishiguro N (2010). Factors influencing death at home in terminally ill cancer patients. Geriatr Gerontol Int.

[73] Kumar SK (2007). Kerala, India: a regional community-based palliative care model. J Pain Symptom Manag.

[74] Kumar S (2013). Models of delivering palliative and end-of-life care in India. Curr Opin Support Palliat Care..

[75] Brazil K, Bedard M, Krueger P, Abernathy T, Lohfeld L, Willison K (2007). Service preferences among family caregivers of the terminally ill. J Palliat med. 2005;8(1):69-77. Zapart S, Kenny P, Hall J, Servis B, Wiley S. home-based palliative care in Sydney, Australia: the carer's perspective on the provision of informal care. Health Soc Care Community..

[76] Burge F, Lawson B, Johnston G, Asada Y, McIntyre PF, Grunfeld E, et al. Bereaved family member perceptions of patient-focused family-centred care during the last 30 days of life using a mortality follow-back survey: Does location matter? BMC Palliat Care. 2014;13(1):25. 10.1186/1472-684X-13-25 PMC4030729 24855451

[77] Milberg A, Strang P, Jakobsson M (2004). Next of kin's experience of powerlessness and helplessness in palliative home care. Support Care Cancer.

[78] Stajduhar KI, Funk L, Toye C, Grande G, Aoun S, Todd C (2010). Part 1: home-based family caregiving at the end of life: a comprehensive review of published quantitative research (1998-2008). Palliat Med.

[79] Meeker MA, Waldrop DP, Schneider J, Case AA (2014). Contending with advanced illness: patient and caregiver perspectives. J Pain Symptom Manag.

[80] Gott M, Moeke Maxwell T, Allen R, Robinson J, Gardiner C (2014). The financial costs of family and whanau caregiving within a palliative care context. Palliat Med.

[81] Moutinho S, Lima L (2014). Family support in palliative care: the relationship between the perceptions of caregivers burden, distress and benefits of caring. Psychooncology..

[82] Mooney K, Berry P, Wong B, Donaldson G (2015). The last 8 weeks of life: family caregiver distress and patient symptoms. J Pain Symptom Manag.

[83] Roche V (2009). The hidden patient: addressing the caregiver. Am J Med Sci.

[84] Wittenberg-Lyles E, Demiris G, Oliver DP, Burt S (2011). Reciprocal suffering: caregiver concerns during hospice care. J Pain Symptom Manag.

[85] Keesing S, Rosenwax L, McNamara B (2011). 'Doubly deprived': a post-death qualitative study of primary carers of people who died in Western Australia. Health Soc Care Community.

[86] Foreva G, Assenova R (2014). Hidden patients: the relatives of patients in need of palliative care. J Palliat Med.

[87] Kahissay MH, Fenta TG, Boon H (2017). Beliefs and perception of ill-health causation: a socio-cultural qualitative study in rural north-eastern Ethiopia. BMC Public Health.

[88] Aarons DE (1999). Medicine and its alternatives: health care priorities in the Caribbean. Hast Cent Rep.

[89] Koffman J, Goddard C, Gao W, Jackson D, Shaw P, Burman R (2015). Exploring meanings of illness causation among those severely affected by multiple sclerosis: a comparative qualitative study of black Caribbean and white British people. BMC Palliat Care.

[90] Landrine H, Klonoff EA (1994). Cultural diversity in causal attributions for illness: the role of the supernatural. J Behav Med.

[91] Logie D, Leng M (2007). Africans die in pain because of fears of opiate addiction. BMJ (Clinical research ed).

[92] García César Amescua, Santos Garcia Joao Batista, Rosario Berenguel Cook María del, Colimon Frantz, Flores Cantisani José Alberto, Guerrero Carlos, Rocío Guillén Núnez María del, Hernández Castro John Jairo, Kraychete Durval Campos, Lara-Solares Argelia, Lech Osvandré, Rico Pazos María Antonieta, Gallegos Manuel Sempértegui, Marcondes Lizandra Pattaro (2018). Undertreatment of pain and low use of opioids in Latin America. Pain Management.

[93] Krakauer EL, Phuong Cham NT, Husain SA, Hai Yen NT, Joranson DE, Khue LN (2015). Toward safe accessibility of opioid pain medicines in Vietnam and other developing countries: a balanced policy method. J Pain Symptom Manag.

[94] de Sola H, Salazar A, Dueñas M, Failde I (2018). Opioids in the treatment of pain. Beliefs, knowledge, and attitudes of the general Spanish population. Identification of subgroups through cluster analysis. J Pain Symptom Manag.

[95] Verloo H, Mpinga EK, Ferreira M, Rapin CH, Chastonay P (2010). Morphinofobia: the situation among the general population and health care professionals in north-eastern Portugal. BMC Palliat Care..

[96] Gunnarsdottir S, Kaasa S, Klepstad P, Sigurdardottir V, Kloke M, Radbruch L (2017). A multicenter study of attitudinal barriers to cancer pain management. Support Care Cancer.

[97] Powe BD, Finnie R (2003). Cancer fatalism: the state of the science. Cancer Nurs.

[98] Lebaron V, Beck SL, Maurer M, Black F, Palat G (2014). An ethnographic study of barriers to cancer pain management and opioid availability in India. Oncologist.

[99] Higginson I, Priest P, McCarthy M (1994). Are bereaved family members a valid proxy for a patient's assessment of dying?. Soc Sci Med.

[100] McPherson CJ, Addington-Hall JM (2003). Judging the quality of care at the end of life: can proxies provide reliable information?. Soc Sci Med.

[101] Tang ST, McCorkle R (2002). Use of family proxies in quality of life research for cancer patients at the end of life: a literature review. Cancer Investig.

[102] Jones JM, McPherson CJ, Zimmermann C, Rodin G, Le LW, Cohen SR (2011). Assessing agreement between terminally ill cancer patients' reports of their quality of life and family caregiver and palliative care physician proxy ratings. J Pain Symptom Manag.

